# Physics and rationale in pediatric abdomen photon counting detector computed tomography: an investigative review towards development of a pediatric abdomen protocol

**DOI:** 10.1007/s00247-025-06274-7

**Published:** 2025-06-11

**Authors:** J Braaksma, MJW Greuter, MV Verhagen

**Affiliations:** Department of Radiology, University Medical Center Groningen, Groningen, 9713GZ Netherlands

**Keywords:** Abdomen, Absorptiometry, Photon, Child, Radiation dosage, Radiology, Signal-to-noise ratio, X-ray computed tomography

## Abstract

The advent of photon counting detector computed tomography (PCD-CT) holds the promise of radiation reduction, improved spatial resolution, and spectral data for material decomposition in pediatric abdominal radiology, but effort is required to achieve this. Consensus lacks on what a good pediatric abdominal protocol should entail, and new features and hardware require a learning curve. This investigative review addresses and investigates the key software and hardware parameters of PCD-CT by testing progressively complex phantoms. Based on these tests, a pediatric abdominal PCD-CT protocol was derived for children up to 12 years, and preliminary clinical results are presented. Pediatric radiologists adopting PCD-CT can benefit from our early experience and use this review to adapt the proposed protocol to their needs.

**Graphical abstract:**

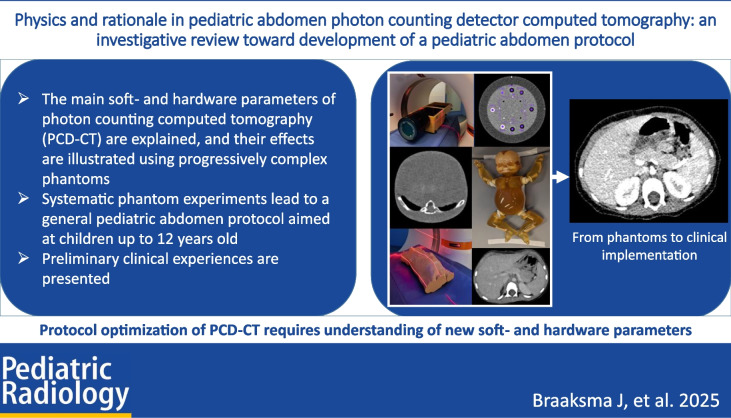

**Supplementary Information:**

The online version contains supplementary material available at 10.1007/s00247-025-06274-7.

## Introduction

Photon counting detector computed tomography (PCD-CT) is a recent innovation in clinical computed tomography (CT) that promises advantages over conventional CT. Whereas conventional CT uses energy integrating detectors that detect light converted from X-ray photons, photon counting detectors (PCD) use semiconductors to directly register charge pairs derived from X-rays [1, 2]. PCDs do not require septa to prevent light scattering, which improves spatial resolution. Furthermore, not only can the incoming X-rays be detected individually, but their energy can also be detected. This allows spectral image post-processing and removal of low energy electrical noise [1, 2]. Subsequently, virtual monoenergetic images (VMI) can be calculated, which represent the dataset as if it had been acquired with one specific photon energy, rather than all the photon energies present in the output spectrum of an X-ray tube. Depending on the clinical indication, technique, and the desired contrast, VMI can range from 40 kiloelectron volt (keV) to 190 keV.

Further reported benefits include improved iodine signal-to-noise ratio and contrast, but also reduced radiation dose [3, 4]. Pediatric patients in particular may benefit. However, new hardware and software parameters in PCD-CT require a new understanding in order to reap the potential benefits. The Siemens NAEOTOM Alpha (Siemens Healthineers, Forchheim, Germany) is the first commercially available, clinically approved PCD-CT. PCD-CTs by other vendors are in pre-clinical or prototype stages [5], and for which no pediatric studies are available. Regarding the Siemens PCD-CT, several helpful reviews have been published [1, 3, 6]. Two papers add value to pediatric abdomen PCD-CT literature. Horst et al. suggested several protocols but substantiated their suggestions with general theory instead of phantom or patient data [2]. Zhou et al. presented a phantom study with the aim of providing a high voltage (120 kV; kilovolt) protocol for children of all sizes, but did not investigate clinical implementation [7]. Furthermore, we observed that neither study sufficiently substantiated a protocol that aligns with the ALARA (as low as reasonably achievable) principle [8]. Therefore, we set out to perform a series of phantom tests aimed at investigating the effects of various PCD-CT parameters, with the aim of developing and subsequently implementing a pediatric abdominal protocol for children up to 12 years (approximately 40 kg) [9].

In this investigative review, we share our acquired insights, provide (pediatric) radiologists with tools to adapt and improve their PCD-CT protocols, provide a pediatric abdominal PCD-CT protocol as a starting point for anyone adopting PCD-CT, and illustrate our preliminary clinical results.

## Quantitative evaluation of image quality using phantoms

**Fig. 1:**
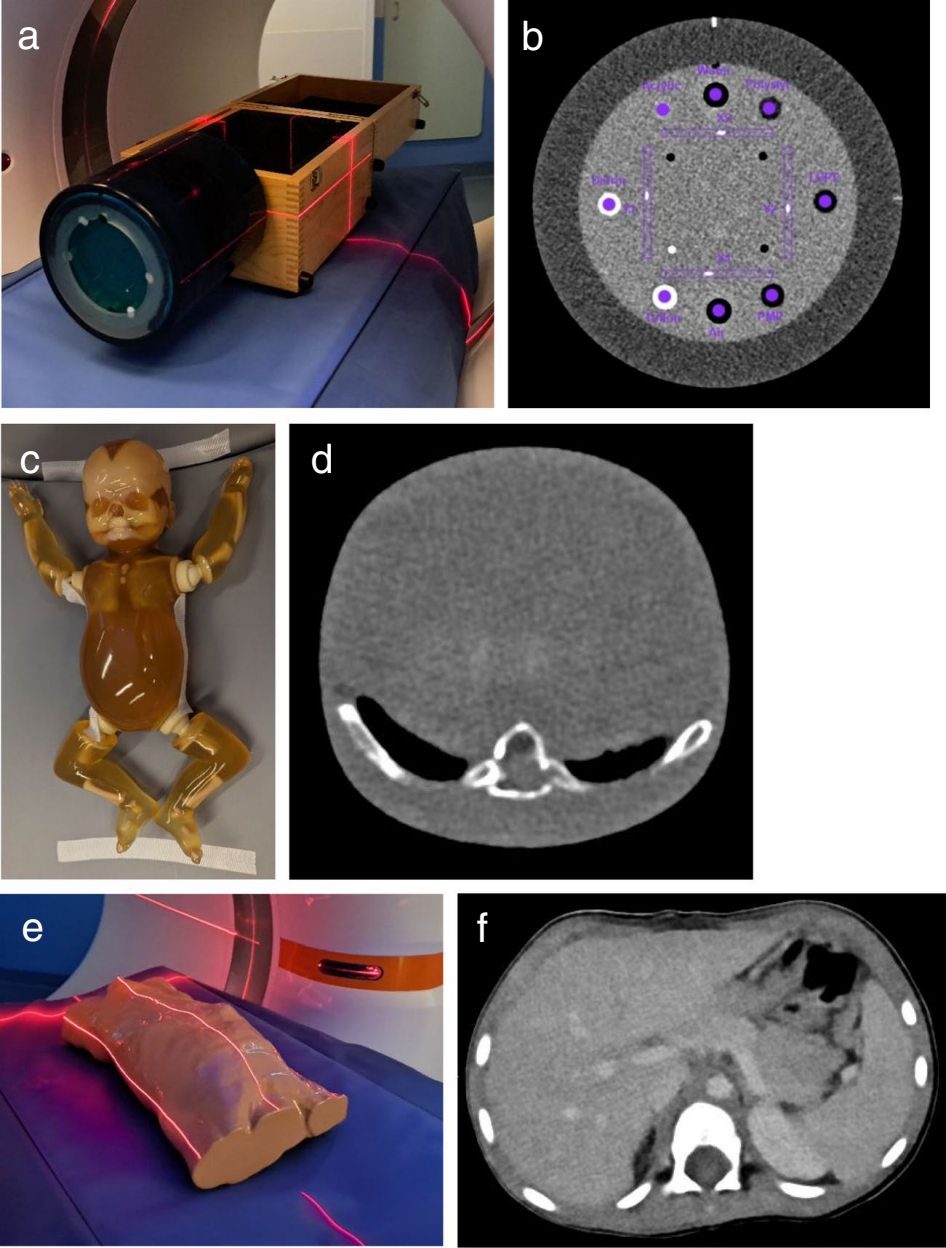
Phantoms used for the presented experiments. **a**, **b** Technical phantom (Catphan 600, Phantom Laboratory, Salem, NY) and an example of a corresponding axial computed tomography (CT) image. **c**, **d** Morphological phantom of a newborn (PBU-80; Newborn Whole Body Phantom, Kyoto Kagaku, Kyoto, Japan) and an example of a corresponding axial CT image. **e**, **f** Phantom of a 3-year-old (Child Torso Phantom PX-58–04, PhantomX, Berlin, Germany) and an example of a corresponding axial CT image

To illustrate and investigate the effects of various PCD-CT settings, a technical phantom (Catphan 600, Phantom Laboratory, Salem, NY) was used for obtaining objective measurement not available in morphological phantoms. Furthermore, a simple morphological phantom of a newborn (PBU-80; Newborn Whole Body Phantom, Kyoto Kagaku, Kyoto, Japan) was used to determine if findings were similar to the technical phantom in a similarly sized morphological phantom. Last, an anatomical morphological phantom of a 3-year-old (PX-58–04; PhantomX, Berlin, Germany) was used to investigate reconstruction parameters prior to clinical implementation. These phantoms did not include simulated pathology. Phantoms underwent PCD-CT scans as detailed in the paragraphs below (Fig. 1).

In this investigative review, relevant PCD-CT parameters are systematically addressed. For each parameter, we start with a brief description of the relevant technology and physics, followed by technical and morphological phantom acquisition and reconstruction parameters designed to better understand and illustrate the workings of PCD-CT, and conclude with an interpretation towards a sound protocol. Next, reconstruction parameters are investigated using the advanced anatomical phantom of a 3-year-old. Last, we present our first clinical results using this protocol, and compare the dose of the obtained protocol to that of conventional CT (SOMATOM Force, Siemens Healthineers, Forchheim, Germany).

Radiation dose is primarily expressed using the volume computed tomography dose index (CTDIvol), measured in milliGray (mGy), and was registered from the PCD-CT console. CTDIvol is a standardized measure of radiation output, protocol-specific, and is the most suitable radiation dose parameter for comparing different protocols in helical CT. The dose-length-product (DLP) is given when pitch is specifically investigated.

Noise is quantified using the standard deviation (SD) of a region of interest (ROI) and is expressed in Hounsfield units (HU) [10, 11]. A higher SD indicates more noise, and noise is strongly affected by tube current (milliampere second, mAs), tube voltage (kV), kernel, and iterative reconstruction. Noise is also investigated using the noise power spectrum (NPS), which quantifies the magnitude of noise as a function of spatial frequency and is expressed in units of HU^2^mm^2^. Peak NPS is a measure for the amount of noise in the image. Last, the contrast to noise ratio (CNR) of liver parenchyma compared to portal enhancement is calculated when possible. CNR is calculated as the CT-value (HU) of the hilar portal vein minus the CT-value of the liver parenchyma, divided by the noise of liver parenchyma (Fig. 1). A higher CNR indicates an improved ability to distinguish a structure from its background in the presence of noise.

For comparing spatial resolution, the modulation transfer function (MTF) is calculated using the 0.28-mm bead in module CTP528 of the Catphan 600 phantom (Phantom Laboratory, Salem, NY). Catphan 600 measurements were semi-automatically analyzed using AutoQA Plus (version 1.8, QA Benchmark, Ellicott City, MD). The 10% value of the MTF is presented (MTF10), which quantifies the transfer of contrast at a specific resolution (the line-pair frequency), and this allows for comparison between techniques. MTF10 is expressed as line-pairs (lp) per cm. A higher MTF10 indicates more line-pairs/cm and higher spatial resolution.

The peak NPS and noise (SD) were obtained from the CTP486 module of the Catphan 600 phantom. Noise was defined at 40% of the phantom diameter. For PBU-80 and PX-58–04, sampled regions of interest (ROI) were obtained, and these were the same for all reconstructed slices (Fig. 1).

Table 1 lists relevant acquisition and reconstruction parameters which are tested in the experiments below, and lists those that were not tested. In the experiments below, if a setting is not specified, the standard setting was used as indicated in Table 1.

**Table 1:** Photon counting computed tomography experimental parameters

Acquisition parameters	
Scan mode and tube voltage, kV	Quantum (70 or 90)^a^ or QuantumPlus (120)
Number of slices, mm	144 × 0.4 (standard)^a^ or 120 × 0.2 (QHD)
Z-coverage, mm	57.6 (standard)^a^ or 24.0 (QHD)
Rotation time, s	0.25
IQ level	100^a^ or 120
Pitch	3.0^a^ (FLASH) or 1.5 (no FLASH)
CARE keV	On
CARE Dose4D	On
DAC	Very weak, weak, average^a^, strong, or very strong
QHD	On or off^a^
Tissue of interest setting	Soft tissue mode with contrast
Reconstruction parameters	
Reconstructed slice thickness, mm	0.4, 1^a^, or 3.0 (0.3, 0.6, or 2.0 increment)
VMI, keV	53^a^, 60, 70, or T3D
Kernels	Br 36, 40^a^, or 44
QIR	1, 2^a^, 3, or 4
Window level/window width	55/335

^a^Standard setting in experiments

*DAC* dose adaption curve, *FLASH* ultra-fast high-pitch, *QHD* quantum high definition, *QIR* quantum iterative reconstructive algorithm, *VMI* virtual monoenergetic image

## Tube voltage selection: Quantum and QuantumPlus mode

In PCD-CT, the tube voltage depends on the selected scan mode: either Quantum or QuantumPlus. In Quantum mode, the tube voltage can be either 70 kV or 90 kV; in QuantumPlus mode, the tube voltage can be either 120 kV or 140 kV. The tube voltage can be selected manually or automatically. When CARE keV is on, the tube voltage is automatically selected based on the size of the patient, as detected on the topogram, within the selected Quantum or QuantumPlus mode. Full use of spectral post-processing, such as virtual non-contrast and iodine maps, is only possible with QuantumPlus mode, whereas Quantum mode is limited to the different energies for VMI reconstruction. Although Quantum mode with its lower tube voltage is expected to yield a lower radiation dose than QuantumPlus mode (at the same tube current), tube current modulation (CARE Dose4D) will automatically modulate tube current during the scan to achieve an approximately constant noise level over the scanned volume of the patient. This noise level is controlled by a preset IQ level (image quality reference mAs) [12]. Therefore, CARE Dose 4D will increase the tube current when the tube voltage is lowered, and vice versa, based on the user-selected IQ level.

To investigate how these two modes impact dose and image quality, Quantum mode (70 kV and 90 kV) and QuantumPlus (120 kV) were compared in the Catphan 600 and PBU-80 phantoms.

In both phantoms, despite CARE Dose4D, CTDIvol increased along with higher kilovolts, with up to 2.1 times higher CTDIvol for 120 kV compared to 70 kV (2.60 mGy versus 1.23 mGy on PBU-80, respectively, Table 2). Noise was mildly lower at 90 kV compared to 70 kV and 120 kV in both phantoms, and peak NPS was also lowest at 90 kV. MTF10 did not vary substantially between kilovolts.

**Table 2:** Effect of tube voltage on image quality parameters

Phantom	Catphan 600			PBU-80		
Scan mode	Q	Q	Q+	Q	Q	Q+
Tube voltage (kV)	70	90	120	70	90	120
IQ level	100	100	100	120	120	120
CTDIvol (mGy)	0.85	1.11	1.29	1.23	2.19	2.60
Noise (SD HU)	22.6	19.0	22.3	15.9	12.4	13.6
Peak NPS (HU^2^mm^2^)	802	565	758	n/a	n/a	n/a
MTF10 (lp/cm)	6.37	6.51	6.17	n/a	n/a	n/a

*CTDIvol*, computed tomography dose index volume, *MTF10*, 10% of modulation transfer function, *n/a* not applicable, *NPS*, peak of noise power spectrum, *SD*, standard deviation, *Q*, Quantum; *Q+*, QuantumPlus

For the purpose of a standard pediatric abdomen protocol, we foremost want the lowest radiation dose, provided that diagnostic image quality is sufficient (as low as reasonably achievable (ALARA)). The lowest tube voltage yields substantially lower dose; while this does result in higher noise, we consider this the starting point for the protocol.

## Virtual monoenergetic image

PCD-CT detects single X-ray photons, but can also measure its effective energy. Dependent on their effective energy, photons are assigned to different energy bins upon detection. In the Alpha PCD-CT two energy bins are currently available: < 50 keV and ≥ 50 keV. From each energy bin, a 3-dimensional dataset is calculated, from which VMI can be reconstructed. In Quantum mode any VMI between 40–130 keV can be reconstructed, whereas in QuantumPlus mode any VMI between 40–190 keV can be reconstructed. Low energy (e.g., 53 keV, as suggested by Siemens) VMI improves soft tissue contrast and iodine contrast by approximating the K-edge of iodine (approximately 33 keV [13]). Conversely, a high energy VMI (e.g., 120 keV) reduces high-density artifacts. It is also possible to produce a full (20–120 keV) spectral image, which is called T3D and approaches conventional CT.

To investigate the effect of VMI on image quality, and how this is dependent on acquisition parameters, the Catphan 600 was scanned at 70 kV, 90 kV, and 120 kV. Subsequently, VMIs at 53 keV, 60 keV, 70 keV, and T3D were reconstructed. For Quantum mode at 70 kV, noise and peak NPS were lowest at T3D, after which 53 keV and 60 keV were preferable over 70 keV; MTF10 did not substantially differ (Table 3). For Quantum mode at 90 kV, VMI at 53 keV and T3D provided the lowest noise and best MTF10, while peak NPS was higher at 53 keV. For QuantumPlus mode at 120 kV, VMI at 70 keV or T3D yielded the lowest noise, with MTF10 similar for all reconstructions.

**Table 3:** Effect of virtual monoenergetic image on image quality parameters

Phantom	Catphan 600											
Scan mode	Q	Q	Q	Q	Q	Q	Q	Q	Q+	Q+	Q+	Q+
Tube voltage (kV)	70	70	70	70	90	90	90	90	120	120	120	120
VMI (keV)	53	60	70	T3D	53	60	70	T3D	53	60	70	T3D
CTDIvol (mGy)	0.85	0.85	0.85	0.85	1.11	1.11	1.11	1.11	1.29	1.29	1.29	1.29
Noise (SD HU)	22.6	22.9	24.1	20.0	19.0	22.9	24.1	20.0	22.3	19.4	16.4	16.1
Peak NPS (HU^2^mm^2^)	802	750	1000	630	565	400	480	410	758	570	390	400
MTF10 (lp/cm)	6.37	6.88	6.13	6.24	6.51	6.20	5.93	6.62	6.17	6.03	6.09	6.08

*CNR*, contrast to noise ratio; *CTDIvol*, computed tomography dose index-volume; *MTF10*, 10% of modulation transfer function; *NPS*, peak of noise power spectrum; *Q*, Quantum; *Q+*, QuantumPlus; *SD*, standard deviation

When scanning in Quantum mode at 70 kV, for our protocol we prefer VMI 53 keV. This combines relatively low radiation dose with low noise, without effect on spatial resolution, and this should provide optimal soft tissue contrast. Of course, multiple VMIs can be reconstructed when clinically warranted.

## IQ level and CARE Dose4D

The IQ level is an image quality adjuster used in PCD-CT which replaces the quality reference mAs used in (Siemens) conventional CT. The CARE Dose4D feature uses the IQ level to determine the effective milliampere-seconds based on changes in patient diameter on the topogram. The dose is linearly dependent on tube current, and therefore also on IQ level.

**Fig. 2:**
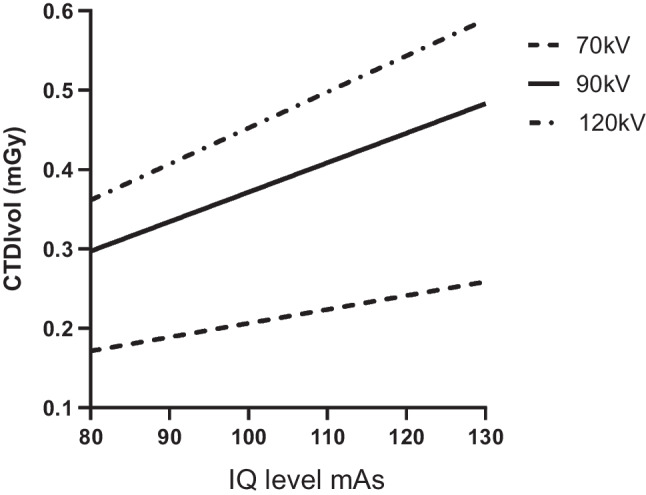
The linear relation between radiation dose and IQ level is illustrated in the PBU-80 (Kyoto Kagaku, Kyoto, Japan) morphological phantom. Both higher IQ level and kV result in increased radiation doses. CTDIvol computed tomography dose index-volume

To confirm this linear relationship, and to illustrate the effect of incremental kilovolts, the PBU-80 phantom underwent scans at incremental IQ levels for Quantum mode at 70 kV, 90 kV, and 120 kV. The linear relation between IQ level and dose is illustrated in Fig. 2.

How much noise is clinically allowed in a pediatric abdomen CT will ultimately vary on the indication for the CT, variables like kernel and iterative reconstruction, and may also be user dependent. There is no definite correct IQ level. Based on our local experience, as also advised by Horst et al. [2], for pediatric abdominal CT we currently employ an IQ level of 120.

## Dose adaption curve and CARE Dose4D

The CARE Dose4D feature modulates tube current not only based on the IQ level, the topogram, and modulation during the scan, but also on the selectable dose adaption curve (DAC) setting. The DAC algorithm dictates to what degree tube current is modulated dependent on the size of the patient compared to a 75-kg reference patient (12). Although DAC is present on all Siemens CTs, it is an important weight dependent dose modulator to investigate for the purposes of this paper. Five DACs are available: very weak, weak, average, strong, and very strong. The DAC curves may appear paradoxical in children. Namely, a strong or very strong setting will substantially increase the dose in a heavy patient compared to the reference patient but will lower the dose in a child. Conversely, a weak or very weak setting will result in a relatively higher dose in children [14], and a relatively lower dose in adults. The DAC system on the Siemens Alpha photon-counting CT is the same as for the conventional Siemens Force CT.

**Fig. 3:**
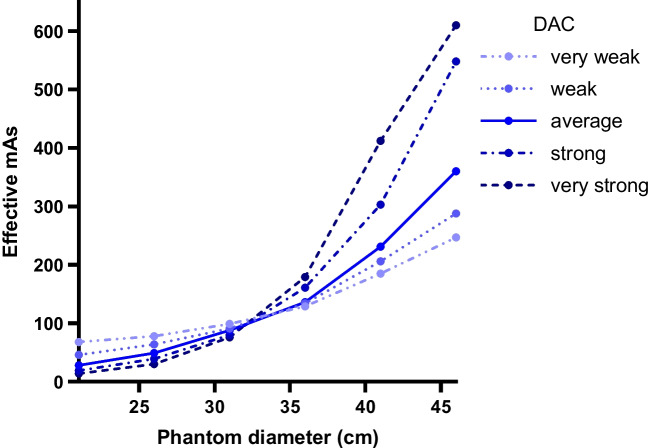
The effect of dose adaption curves (DAC) on the effective milliampere-seconds in progressively larger phantoms on conventional computed tomography (SOMATOM Force, Siemens Healthineers, Forchheim, Germany). DAC “strong” and “very strong” result in relatively higher doses in large patients, and fewer doses in small patients

**Fig. 4:**
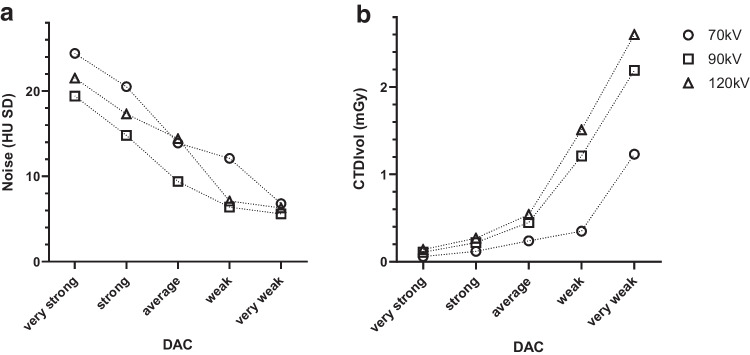
The effect of dose adaption curves (DAC) on noise (**a**) and CTDIvol (computed tomography dose index-volume, **b **at incremental tube voltages in a newborn phantom (PBU-80, Kyoto Kagaku, Kyoto, Japan). Noise decreases approximately linearly with increasing DAC; radiation dose increases exponentially with increasing DAC. HU Hounsfield unit, SD standard deviation. The datapoints of these graphs are given in Supplementary Material 1

Figure 3 illustrates the effect of the DAC setting on effective milliampere-seconds in progressively larger diameter phantoms. To further determine the effect of the DAC setting and tube voltage on dose and noise, we also scanned the PBU-80 phantom (diameter 10.7 cm) for all 5 DAC settings for Quantum mode at 70, 90, and 120 kV (Fig. 4).

There was a steep increase of dose for DAC very weak at all tube voltages, and a substantial increase for DAC weak at 90 kV and 120 kV (Fig. 4). For dose at 70 kV this varied by a factor of 20.5 (ranging from 0.06 mGy to 1.23 mGy between very strong and very weak, respectively). In contrast, noise decreased largely linearly (Supplementary Material 1, Fig. 4). Ultimately, DAC choice depends on the goal; DAC average gives a more equal image quality over different size patients, while DAC strong brings further dose reduction for the lighter, most radiation sensitive patients at the cost of more noise. For the first iteration of our clinical protocol, we chose DAC average.

## Ultra-fast high-pitch mode

The ultra-fast high-pitch (FLASH) protocol with pitch up to 3.2 is provided by the dual source PCD-CT Alpha [15]. The FLASH protocol shortens scanning time significantly, so that movement artifacts in crying babies may be absent or negligible [16, 17]. However, the high pitch means that interpolation artifacts might occur [18]. Because in FLASH mode less data is available for each slice, without CARE Dose4D, noise is expected to be higher for FLASH mode than without. However, with CARE Dose4D always on, this should keep noise stable by increasing mA. Furthermore, because of overranging, the DLP will increase with pitch while CTDIvol will stay constant at equal kV [19]. Although overranging is partially dynamically shielded to minimize the dose penalty, this dose increase of the FLASH protocol should be taken into account.

The Catphan 600 phantom was scanned with and without the FLASH protocol. Both dose (DLP) and noise were slightly higher with the FLASH protocol. Scan time was 1.72 times longer without FLASH mode (0.74/0.43 s, Table 4).

**Table 4:** Effect on image quality and dose of increased pitch resulting from the ultra-fast high-pitch protocol

Phantom	Catphan 600	
Scan mode	FLASH	No FLASH
Pitch	3.0	1.5
Scan time (s)	0.43	0.74
CTDIvol (mGy)	0.66	0.67
DLP (mGy × cm)	17.3	15.1
Noise (SD HU)	22.6	20.8
Peak NPS (HU^2^mm^2^)	802	601
MTF10 (lp/cm)	6.37	6.15

*CTDIvol* computed tomography dose index-volume, *DLP* dose-length-product, *FLASH* ultra-fast high-pitch protocol, *MTF10* 10% of modulation transfer function, *NPS* peak of noise power spectrum, *SD* standard deviation

The need for reduced movement artifacts must be weighed against potential interpolation artifacts, higher dose, and higher noise of the FLASH protocol. Consequently, in a pediatric abdominal CT, we suggest that FLASH should only be used in children unable to hold their breath for the duration of the scan. As a guideline, children at 6 years old can generally breath-hold for the duration of the scan [20].

## Resolution and Quantum high definition

The solid-state photon counting detector directly detects individual X-ray photons, obviating the need for detector septa, and this results in smaller detector pixels than conventional CT. The PCD-CT subpixels measure 0.151 mm × 0.176 mm at the isocenter [21]. For standard imaging, 2 × 2 subpixels are binned at a pixel size of 0.302 mm × 0.352 mm. For ultra-high resolution imaging (QHD (Quantum high definition)) the individual subpixels can also be used. This results in an in-plane resolution of up to 0.208 mm and 0.125 mm for standard and QHD mode, respectively [15]. Minimum slice thickness (ST) may be 0.4 mm for standard imaging and 0.2 mm for QHD [15]. In comparison, conventional CT is generally limited to 0.5–0.6 mm ST. For QHD, the detector width is reduced from 144 × 0.4 mm = 57.6 mm to 120 × 0.2 mm = 24.0 mm, which increases the scan time, and this may result in more motion artifacts.

Optimal use of available resolution is dependent on the field of view (FOV) and the reconstruction matrix size. The matrix size can be set at 512 × 512 pixels, 768 × 768 pixels, or 1024 × 1024 pixels [15]. Supplementary Material 2 illustrates how FOV and matrix size determine the minimal pixel size and thereby maximal resolution. Minimal pixel size is limited by the maximal in-plane resolution of the detector, and increasing the matrix or decreasing the FOV beyond this would not improve resolution. Furthermore, a bigger matrix will result in smaller pixels, and subsequently more noise, which would warrant more dose. Based on the necessary FOV and the reconstruction kernel, the Alpha PCD-CT can automatically adjust for the optimal matrix size.

To investigate the effects of QHD mode, we compared Quantum and QuantumPlus scans with and without QHD on the Catphan 600. For QHD scans, a pediatric chest protocol was applied because the PCD-CT Alpha does not provide a QHD protocol for pediatric abdomen.

QHD scans yielded higher doses at all tube voltages, and with less noise and peak NPS (Table 5). This higher dose may be the result of the smaller width of the detector in QHD scanning, resulting in more X-ray beam penumbra. The effect of this will vary depending on the length of the scan. However, it may be achievable to reach lower doses by lowering the IQ level, which would then increase noise. Further experiments are necessary to investigate this. Last, QHD scan times were twice as long as standard scans, and MTF10 did not vary substantially at 1-mm reconstructions. For the purpose of abdominal CT, ST 1 mm will generally be sufficient, and potential increased motion artifacts resulting from the doubled scan time make QHD mode unfavorable artifact. However, this may be different if there is a clinical indication to obtain thinner slices, and this may also be a focus for further research.

**Table 5:** Quantum high definition (all 1-mm reconstructions)

Phantom	Catphan 600					
Scan mode	Standard Q70	QHD Q70	Standard Q90	QHD Q90	Standard Q + 120	QHD Q + 120
CTDIvol	0.85	1.17	1.11	1.53	1.29	2.24
Noise (SD HU)	22.6	18.8	19	16.4	22.3	13.6
Scan time (seconds)	0.44	0.88	0.44	0.88	0.44	0.88
Peak NPS HU^2^mm^2^	802	576	565	441	758	298
MTF10	6.37	6.12	6.51	5.98	6.17	6.22

*CTDIvol* computed tomography dose index-volume, *MTF10* 10% of modulation transfer function, *NPS* peak of noise power spectrum, *Q* Quantum, *Q* + QuantumPlus, *SD* standard deviation

## Kernel, slice thickness, and iterative reconstruction

For abdomen, the body regular (Br) kernel is indicated, ranging from Br36 to Br98. The number indicates the resolution index, a higher index results in a higher spatial resolution, but also higher noise [22]. In addition, the body vascular (Bv) kernels can be applied to reduce blooming artifacts of high attenuation structures like vessels with iodine. Kernels of 80 and higher are reserved for QHD imaging. Iterative reconstruction (quantum iterative reconstruction (QIR)) ranges in strength from 1 to 4, with 4 being the strongest [23, 24].

To determine the impact of post-processing variables kernel, ST, and QIR strength on radiation dose and image quality variables, we tested the anatomical phantom of a 3-year-old (PX-58–04).

As expected, thicker slices decrease noise and increase CNR (Table 6). Of note, if a slice thickness of 3 mm is clinically acceptable, radiation dose could be lowered to keep noise stable compared to 1 mm. Softer Br kernels result in lower noise and higher CNR, and higher QIR lower noise and improve CNR (Table 6). However, soft Br kernels also decrease spatial resolution, and for our protocol we prefer Br40. Higher QIR levels decrease noise or can be used to decrease dose while keeping noise stable, for which it is an established and powerful tool. Depending on the type of iterative reconstruction, reported disadvantages of higher iterative reconstruction strength levels are noise texture differences, and blurred or cartoon-like images [25].

**Table 6:** Effects of slice thickness, kernel and iterative reconstruction on image quality

Phantom	PX-58-04									
Slice thickness (mm)	0.4	1	3	1	1	1	3	3	3	3
Kernel	Br 36	Br 36	Br 36	Br36	Br40	Br44	Br40	Br40	Br40	Br40
QIR	2	2	2	2	2	2	1	2	3	4
CTDIvol (mGy)	0.60	0.60	0.60	0.60	0.60	0.60	0.60	0.60	0.60	0.60
Noise (SD HU) of liver parenchyma	31	20	12	20	25	32	19	17	14	12
CT-number (HU) of liver parenchyma	145	153	148	153	154	154	152	151	151	151
CNR	1.91	2.12	4.27	2.12	1.66	1.34	2.34	2.73	3.25	3.97

*Br*, body reconstruction kernel; *CNR*, contrast to noise ratio; *CTDIvol*, computed tomography dose index volume; *QIR*, quantum iterative reconstruction algorithm

## Clinical application and advice

**Fig. 5:**
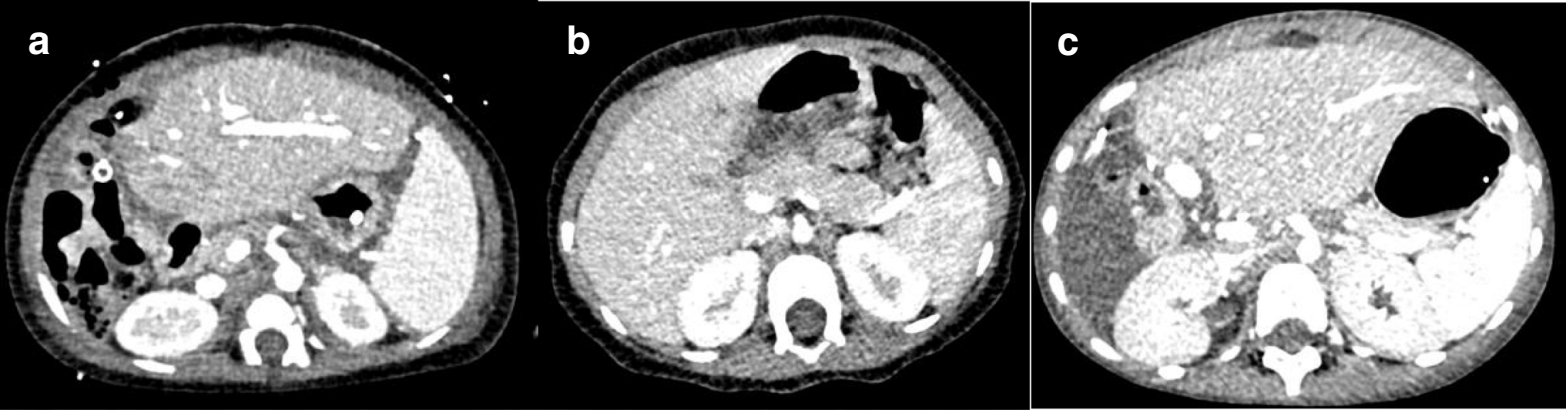
Examples of the clinical implementation of the pediatric abdomen protocol. Axial computed tomography slices in the portal venous phase after intravenous contrast demonstrate the appearances of contrast and noise of the protocol at different ages. Protocol: 70 kV Quantum mode at 53 keV virtual monoenergetic image, IQ level 120, body reconstruction kernel 40, quantum iterative reconstruction 2, 1.0-mm slice thickness. **a** Six-month-old girl after liver transplantation. **b** Two-year-old girl prior to liver transplantation. **c** Six-year-old boy after liver transplantation

**Fig. 6:**
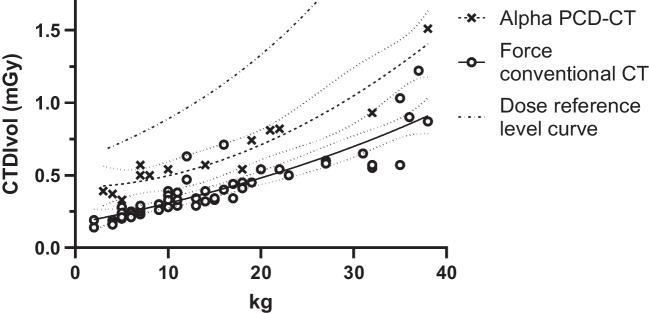
Computed tomography dose index-volume (CTDIvol) in clinical pediatric abdominal computed tomography (CT) as a function of patient weight. Comparison between photon-counting CT and conventional CT (NAETOM Alpha and NAETOM Force, respectively, both from Siemens Healthineers, Forchheim, Germany). Scatterplot with nonlinear regression and 95% confidence intervals, with the national dose reference levels given [27]. Using the current settings, these preliminary data, at this site, and without objective assessment of image quality, show that radiation dose is higher in photon counting detector-CT compared to conventional CT

Based on the provided phantom tests, we constructed and clinically implemented a standard pediatric abdomen protocol (Table 7). This protocol provides clinically acceptable image quality and has been designed in accordance with the ALARA principle (Fig. 5). Our intravenous contrast protocol is noted in Supplementary Material 3. Dose levels of our first PCD-CT scans are illustrated in Fig. 6 and compared to a conventional CT (protocol given in Supplementary Material 4). PCD-CT doses were well below diagnostic reference levels and achievable dose levels [26, 27]. Nevertheless, the dose was substantially higher than those on the conventional CT, with subjectively similar image quality. For example, at 14 kg in two different children, CTDIvol was 0.75 mGy and 0.39 mGy for PCD-CT and conventional CT, respectively. Furthermore, the dose was relatively higher in the youngest children.

**Table 7:** Photon counting detector computed tomography pediatric abdomen protocol

Protocol	Standard	Complex (not used in the presented clinical data, aimed at reduced noise and thinner slices)
Acquisition parameters		
Scan mode	Quantum	QuantumPlus
Siemens protocol	RoutineFlashSpiralChildAbdomenQuantum	RoutineFlashSpiralChildAbdomenQuantumplus
Tube voltage, kV	70/90	120/140
Number of slices, mm	144 × 0.4	144 × 0.4
Z-coverage, mm	58	58
Rotation time, s	0.25	0.25
IQ level	120	120
Pitch	3.2 (FLASH) or 1.5	3.2 (FLASH) or 1.5
Care keV	On	On
CARE Dose4D	On	On
Dose adaption curve	Average	Average
Tissue of interest setting	Soft tissue with contrast	Soft tissue with contrast
Reconstruction parameters		
Reconstructed slice thickness, mm	1.0 and 3.0 (0.6 and 2.0 increment)	0.4, 1.0 and 3.0 (0.3, 0.6 and 2.0 increment)
Matrix	Auto	Auto
VMI, keV	53	53 (T3D for non-contrast if needed)
Kernels	Br40 (1 mm), Br40 (3 mm)	Br40 (1 mm), Br40 (3 mm), Bv60 (0.4, 1 mm)
QIR	2	2
Window level/window width	55/335	55/335
Spectral analysis	No	Iodine map, virtual non-contrast
Intravenous iodine contrast		
Contrast phase	Portal venous, delay is age dependent	Portal venous, delay is age dependent
Volume and flow rate	Age and kV dependent^a^	Age and kV dependent^a^

*Br* body reconstruction kernel, *Bv* body vascular reconstruction kernel, *FLASH* ultra-fast high-pitch protocol, *QIR* quantum iterative reconstruction algorithm, *VMI* virtual monoenergetic image

^a^See Supplementary Material 3

Phantom tests only go so far, and after clinical implementation the user should consider further adaption based on required image quality and post-processing, and radiation dose. If the radiologist experiences too much noise, we suggest to first adapt reconstruction parameters: increase QIR strength, choose a softer kernel, and change window/level settings according to preference. After this, adaption of acquisition parameters can be considered. IQ level will have a linear effect on dose, whereas the DAC will have more impact depending on patient size. Alternatively, scanning in QuantumPlus mode will systematically increase dose and image quality.

## Discussion and future perspectives

This investigative review aimed to provide an improved understanding of the first commercially available and clinically approved PCD-CT by addressing the most relevant physics, and soft- and hardware parameters. These were illustrated with progressively complex phantoms, and this resulted in a sound pediatric abdomen protocol for the PCD-CT aimed at children up to 12 years old (approximately 40 kg). Subsequently, the first clinical results are shown.

PCD-CT Quantum mode offers VMI without extra radiation dose, and QuantumPlus mode adds simultaneous acquisition of spectral data at the cost of increased radiation dose. However, for standard pediatric abdominal imaging, our analysis suggests that standard Quantum mode aligns best with the ALARA principle. This is in contrast to the protocol suggested by Zhou et al., which advocated a QuantumPlus 120-kV protocol for all children, but would systematically overexpose children [28]. Nevertheless, for some indications, reduced noise or spectral analysis may be relevant, which may require scanning in QuantumPlus mode, and this requires further research into this new generation of CT technology. Applications (including potential future applications) of PCD-CT in the pediatric abdomen are discussed below and summarized in Supplementary Material 5.

Preliminary studies suggested that PCD-CT will reduce radiation dose compared to conventional CT, and this was shown for congenital heart CT [3, 29]. However, although our protocol achieves radiation doses well below reference values [26, 27], preliminary doses of the PCD-CT protocol were higher than our more established conventional CT protocol. Radiation dose can easily be decreased by lowering IQ level and adapting the DAC level. Based on our preliminary clinical results, with relatively higher doses in the smallest children, it may be prudent to change DAC from average to strong. However, the aim remains to be diagnostic, and decreasing radiation dose should be carefully monitored. Further research into optimization of the presented pediatric abdomen protocol should include objective multi-reader radiologist assessments for image quality and diagnostic performance. Given the increased contrast of PCD-CT, it may also be possible to accept more noise while keeping diagnostic performance stable. Furthermore, when reducing dose, subsequent increased noise can be corrected with higher QIR and deep learning reconstruction techniques (DLR) [30]. A recent study in adults reported that in PCD-CT, QIR 4 performed best for quantitative and qualitative image quality [23]. Future research may aim to replicate these promising QIR results, and investigate if the same is true in children. Although DLR is not yet available on this PCD-CT, this will become an important topic for future research. For now, pediatric abdominal PCD-CT does not readily reduce dose compared to conventional CT.

Whereas previous dual-energy CTs could suffer from a mismatch between the iodine map and CT images, PCD-CT resolves this by simultaneously obtaining both datasets. Furthermore, spectral data can now be obtained while using the FLASH protocol [31]. However, apart from VMI, acquiring this spectral data does imply scanning in QuantumPlus mode, which entails a higher radiation dose compared to Quantum mode. For the lungs, spectral analysis can provide a pulmonary perfusion map, and this can help to identify peripheral perfusion defects. For the abdomen, the iodine map may improve bowel wall assessment, and lesion detection and characterization [3, 31]. Spectral analysis can be used to characterize renal stones, and the virtual none contrast map may help with stone detection and assess intrinsically high-density structures in the absence of a non-contrast phase. However, it is unclear if these advantages justify systematically performing a higher dose PCD-CT in QuantumPlus mode.

QHD indications are more apparent in coronary, musculoskeletal, and mastoid than abdominal imaging [3]. For the pediatric abdomen, standard PCD-CT at an ST of 1.0 mm will generally be clinically sufficient, and 0.4 mm already appears sufficient to study tiny vessels. Nevertheless, perhaps QHD at 0.2 mm may in some cases replace angiography, and if test accuracy is comparable in combination with a similar radiation dose to angiography, this could prevent unnecessary invasive procedures. This will require further investigation.

Optimal VMI will depend on the clinical indication. Low VMI (40–55 keV) may improve assessment of vessels and focal liver lesions [1, 32, 33], whereas higher VMIs help to reduce noise and artifacts [7]. Because low VMIs approach the iodine K-edge, and consequently improve iodine contrast, the volume of iodine contrast may also be reduced [34]. This also creates opportunities for an improved implementation of split-bolus protocols with reduced contrast. To what extent current contrast protocols can be adapted with optimal use of VMI will require further research.

Determining and deciding what a good pediatric abdominal CT protocol should entail is challenging. Tests on phantoms will be necessary, but extrapolation to patients may not necessarily be optimal. With the many acquisition and reconstruction parameters of PCD-CT, there are many different combinations and possibilities, and there may be different combinations to reach equally valid protocols. Furthermore, in the process of determining a good protocol, decisions regarding image reconstruction and the required level of image quality may be site and disease specific.

In conclusion, in this investigative review, we illustrate the key soft- and hardware parameters of PCD-CT using progressively complex phantoms, from which we derive a general pediatric abdomen protocol aimed for children up to 12 years old. The preliminary clinical results of this protocol are presented, and these, along with potential future applications and research topics, are discussed. We present this paper with the aim of guiding any radiologist engaging with PCD-CT, and to help them adapt the presented protocol to their clinical needs.

## Supplementary Information

Below is the link to the electronic supplementary material.

ESM 1 (DOCX 43.3 KB)

## Data Availability

No datasets were generated or analysed during the current study.
