# Supplementary material for: Physics and rationale in pediatric abdomen photon counting detector computed tomography: an investigative review towards development of a pediatric abdomen protocol
Source: Pediatr Radiol. 2025 Jun 11;55(8):1556–67. doi: 10.1007/s00247-025-06274-7 (PMC12321945; doi:10.1007/s00247-025-06274-7)
Supplement: Supplementary file 1 — (DOCX 43.3 KB) [file 247_2025_6274_MOESM1_ESM.docx]

| Supplementary Material 1: Effect of the dose adaption curves settings (very weak, weak, average, strong and very strong) on computed tomography dose index-volume and noise at different scan modes | | | | | | |
| --- | --- | --- | --- | --- | --- | --- |
| Phantom | PBU-80 | | | | | |
|  | CTDIvol (mGy) | | | Noise (SD HU) | | |
| Scan mode | Q | | Q+ | Q | | Q+ |
| Tube voltage (kV) | 70 | 90 | 120 | 70 | 90 | 120 |
| Very weak | 1.23 | 2.19 | 2.60 | 6.8 | 5.6 | 6.3 |
| Weak | 0.35 | 1.21 | 1.51 | 12.1 | 6.4 | 7.1 |
| AVerage | 0.24 | 0.45 | 0.54 | 13.9 | 9.4 | 14.4 |
| Strong | 0.12 | 0.22 | 0.27 | 20.5 | 14.8 | 17.3 |
| Very strong | 0.06 | 0.11 | 0.14 | 24.4 | 19.4 | 21.5 |
| *CTDIvol* computed tomography dose index-volume, *Q* Quantum; *Q+* QuantumPlus, *SD* standard deviation | | | | | | |

| Supplementary Material 2. Maximum spatial resolution (in mm) as a function of field of view and matrix size | | | |
| --- | --- | --- | --- |
| Matrix | FOV 100 mm | FOV 25 0mm | FOV 500 mm |
| 512*512 | 0.20 | 0.49 | 0.98 |
| 768*768 | 0.13 | 0.33 | 0.65 |
| 1024*1024 | 0.10 | 0.24 | 0.49 |
| *FOV* field of view | | | |

| Supplementary Material 3. Paediatric abdominal computed tomography contrast protocol, up to 40 kg. Volume of contrast (Iomeron 350 mg I/ml), flow rate and delay for weight | | | |
| --- | --- | --- | --- |
| Weight (kg) | Contrast (ml) | Flow rate (ml/seconds) | Delay (seconds) |
| 2 | 5 | Manual injection | 20 |
| 4 | 8 | 1 | 30 |
| 6 | 12 | 1 | 37 |
| 8 | 16 | 1 | 41 |
| 10 | 20 | 1.5 | 45 |
| 12 | 24 | 1.5 | 48 |
| 14 | 28 | 1.5 | 51 |
| 16 | 32 | 1.5 | 53 |
| 18 | 36 | 1.5 | 55 |
| 20 | 40 | 2 | 57 |
| 22 | 44 | 2 | 58 |
| 24 | 48 | 2 | 60 |
| 26 | 52 | 2 | 62 |
| 28 | 56 | 2 | 64 |
| 30 | 60 | 2.5 | 66 |
| 32 | 64 | 2.5 | 68 |
| 34 | 68 | 2.5 | 69 |
| 36 | 72 | 2.5 | 70 |
| 38 | 76 | 2.5 | 71 |
| 40 | 80 | 3 | 72 |
| Contrast dilution. Volumes are based on 100kV. Depending on the clinical indication, contrast may be diluted if scanned at lower kVs:   - 70 kV: 70% contrast, 30% saline - 80 kV: 80% contrast, 20% saline - 90 kV: 90% contrast, 10% saline   The saline flush is dependent on the length of the tube between power injector and intravenous catheter with an extra margin. The flush volume we use is 0.5 x contrast ml, with a minimum of 13 ml and maximum of 32 ml | | | |

| Supplementary Material 4. Standard conventional computed tomography pediatric abdomen protocol (SOMATOM Force, Siemens Healthineers, Forchheim, Germany) | |
| --- | --- |
| Acquisition parameters | |
| Tube voltage, kV | 70 or 90 (based on CARE kV) |
| Collimation, mm | 192 x 0.6 |
| Z-coverage, mm | 58 |
| Rotation time, s | 0.25 |
| Quality reference mAs | 90 |
| Pitch | 3.2 (FLASH) or 1.5 |
| Force kV adaption | On |
| CARE keV | On |
| CARE Dose4D | On |
| Dose adaption curve | Average |
| Tissue of interest (slider 1-12) | 7 |
| Reconstruction parameters | |
| Reconstructed slice thickness, mm | 1 and 3 , with 0.6 and 2mm increment |
| Matrix | Auto |
| Kernels | Br36 |
| Iterative reconstruction, ADMIRE | 1 |
| Window level/window width | 55/335 |
| Intravenous iodine contrast |  |
| Contrast phase | Portal venous, delay is age dependent |
| Volume and flow rate | Age and kV dependent ^a^ |
| *ADMIRE* advanced modeled iterative reconstruction*, Br* body reconstruction kernel*, FLASH* ultra-fast high-pitch protocol, *QIR* quantum iterative reconstruction algorithm, *VMI* virtual monoenergetic image ^a^ see supplementary material 3 | |

| Supplementary Material 5. (potential future) applications of photon counting detector computed tomography in pediatric abdomen | |
| --- | --- |
| **Spectral data with the advantage of simultaneous acquisition** | |
| Virtual non-contrast (Q+) | For multiphase indications, the non-contrast phase can be obtained from a contrast phase, thus removing a scanning phase and reducing radiation dose |
| Iodine map (Q+) | Differentiate enhancement versus intrinsic high density (e.g. to differentiate hemorrhage from tumor[1]) |
| Material separation (not currently available on PCD-CT, Q+) | Fat maps and content quantification [1] |
| Characterize renal stones (Q+) |  |
| VMI (Q, Q+) | Low VMI optimizes soft-tissue contrast and improves Iodine contrast, while high VMI helps to reduce high density artefacts [2]. Detection and characterization of lesions may be improved, and assessment of bowel wall improved [3, 4]. |
| Elimination of electronic noise (Q, Q+) | <25keV can be eliminated from the spectral data, reducing noise, with particular advantage for low tube voltage scans |
| **Improved Iodine contrast** | |
| Reduce contrast (Q, Q+) | With low VMIs the iodine k-edge is approached, improving iodine contrast and allowing for contrast volume reduction [5, 6] |
| Improved split bolus protocol with reduced contrast (Q, Q+) | Improved iodine contrast may make image quality of split bolus protocol better, whilst reducing iodine volume |
| Improved SNR and CNR may allow for more noise or lower dose whilst maintain diagnostic accuracy (Q, Q+) | Multi-reader studies with objective and subjective image quality parameters may find that dose reduction is possible despite higher noise |
| **Higher resolution and thinner slices** |  |
| 0.4 mm slice thickness | Small vessel complications (e.g. hepatic artery anastomotic stenosis after liver transplant) can be assessed with superior resolution, without prolonging the scan time and risking movement artefacts [4] |
| QHD 0.2mm slice thickness | With up to 0.151 mm x 0.176 mm pixel size, angiography grade images may replace invasive angiography |
| **Potentially improved reconstruction parameters** | |
| QIR | A study in adults showed that maximum QIR was superior, this may be true in children as well [7] |
| Bv kernel | Bv kernels reduce blooming artefacts of vessels, improving resolution |
| *Bv* body vascular reconstruction kernel, *CNR* contrast to noise ratio, *PCD-CT* photon countin detector computed tomography, *Q* Quantum mode, *Q+* QuantumPlus mode, *QHD* Quantum high definition, *QIR* Quantum iterative reconstruction, *SNR* signal to noise ratio, *VMI* virtual monoenergetic image | |

References

1. Kamps SE, Otjen JP, Stanescu AL, Mileto A, Lee EY, Phillips GS (2020) Dual-Energy CT of Pediatric Abdominal Oncology Imaging: Private Tour of New Applications of CT Technology. AJR Am J Roentgenol 214:967-975.

2. Sato E, Oda Y, Abudurexiti A, Hagiwara O, Matsukiyo H, Osawa A, Enomoto T, Watanabe M, Kusachi S, Sato S, Ogawa A, Onagawa J (2012) Demonstration of enhanced iodine K-edge imaging using an energy-dispersive X-ray computed tomography system with a 25 mm/s-scan linear cadmium telluride detector and a single comparator. Appl Radiat Isot 70:831-836.

3. Siegel MJ, Ramirez-Giraldo JC (2019) Dual-Energy CT in Children: Imaging Algorithms and Clinical Applications. Radiology 291:286-297.

4. Aliukonyte I, Caudri D, Booij R, van Straten M, Dijkshoorn ML, Budde RPJ, Oei EHG, Saba L, Tiddens H, Ciet P (2024) Unlocking the potential of photon counting detector CT for paediatric imaging: a pictorial essay. BJR Open 6:tzae015.

5. Sawall S, Klein L, Amato C, Wehrse E, Dorn S, Maier J, Heinze S, Schlemmer HP, Ziener CH, Uhrig M, Kachelriess M (2020) Iodine contrast-to-noise ratio improvement at unit dose and contrast media volume reduction in whole-body photon-counting CT. Eur J Radiol 126:108909.

6. Horst KK, Yu L, McCollough CH, Esquivel A, Thorne JE, Rajiah PS, Baffour F, Hull NC, Weber NM, Thacker PG, Thomas KB, Binkovitz LA, Guerin JB, Fletcher JG (2023) Potential benefits of photon counting detector computed tomography in pediatric imaging. Br J Radiol 96:20230189.

7. Sartoretti T, Wildberger JE, Flohr T, Alkadhi H (2023) Photon-counting detector CT: early clinical experience review. Br J Radiol 96:20220544.
